# Assessing Patient-Reported Outcomes: A Mixed Methods Qualitative Comparison Between Obturator and Surgically Reconstructed Maxillectomy Patients

**DOI:** 10.1177/00034894251320003

**Published:** 2025-02-13

**Authors:** Ala Almanaseer, Cecilia Dong, Freeman Paczkowski, Francisco Laxague, S Danielle Macneil, Anthony C. Nichols, John Yoo, Kevin Fung, Cecilia Aragon, Adrian Mendez

**Affiliations:** 1 Schulich School of Medicine & Dentistry, Western University, London, ON, Canada; 2 Department of Otolaryngology—Head and Neck Surgery, London Health and Sciences Center, London, ON, Canada

**Keywords:** maxillectomy, rehabilitation, surgical reconstruction, obturator insertion, functional domains, patient-reported-outcomes

## Abstract

**Objectives:**

Cancers of the maxillary region are often treated surgically with a maxillectomy followed by rehabilitation involving surgical reconstruction or obturator insertion to improve functional outcomes. However, there is a lack of consensus regarding the specific indications for either rehabilitation method. The objective of this study was to identify unique functional domains for maxillectomy patients who underwent surgical reconstruction or obturator insertion post-op to provide standardized data that can inform selection of either method.

**Methods:**

This mixed-methods qualitative research was conducted from January 2020 to June 2022 at London Health Sciences Centre, a tertiary care center in London, Ontario, Canada. Phase I included open-ended patient interviews through grounded theory, while phase II incorporated focus groups through the Delphi technique. Phase I identified functional domains of interest, which were further refined based on importance to patients in phase II. Inclusion criteria consisted of adults, 18 years or older who underwent maxillectomy surgery for head and neck cancer.

**Results:**

A total of 22 patients were included in phase I and 8 patients were included in phase II. The top 4 functional domains that affected patients regardless of rehabilitation method were eating difficulties, speaking difficulties, social discomfort with public eating, and numbness. The top 4 unique functional domains identified for the surgical group were dry mouth, trismus, chewing difficulties, and eye-related symptoms. The top 4 unique functional domains for the obturator group were obturator discomfort, nasal regurgitation, weight loss, and voice changes.

**Conclusions:**

This study identified functional domains affecting maxillectomy patients, which can be used to inform decisions regarding selection of rehabilitation method in clinical practice. This data can also in the future to create the first patient-reported outcomes instrument for this patient population.

## Introduction

In the field of head and neck oncology, cancers of the maxillary region are commonly treated with a maxillectomy surgery.^1^ The function of patients undergoing a maxillectomy can significantly be impacted due to the sensitivity and complexity of the surrounding anatomy.^2^ Functional outcomes of patients can be improved through rehabilitation using surgical reconstruction or obturator insertion.^3^ Each reconstructive procedure has its own set of advantages, with the surgical team ultimately guiding the patient on the final rehabilitation choice based on the extent of the defect and the need for continued therapy.^4^

Identifying domains of interest for patients who have underwent a maxillectomy with either surgical reconstruction or obturator placement can help distinguish key differences in rehabilitation methods. This information can be used to educate patients on which reconstructive technique may be more appropriate for their specific needs and can also be used to create the first patient-reported outcomes (PRO) instrument for maxillectomy patients.

Over the last several years, there has been an increased focus on patient-centered treatment that allows patients to play an important role in their treatment course and decision making.^5^ Patient-reported outcomes (PRO) offer a method of readily identifying patient concerns that cannot be measured using traditional outcomes. Once created, PRO instruments offer a widely accessible and economic tool for clinicians to incorporate patient’s preferences into their treatment plan.^5^

Currently, there is no gold standard method for measuring functional outcomes in head and neck cancer patients undergoing maxillectomy. There are several instruments in the head and neck cancer literature that assist in treatment planning and prognosis.^6,7^ However, the choice of rehabilitation method is largely based on individual preferences, and as such there is a lack of consensus regarding the indication for each rehabilitation method.^1^

The aim of this study is to identify the most important functional areas of concern for patients undergoing maxillectomy surgery and to compare any similarities or differences in these domains between the surgically reconstructed patients and patients provided with an obturator. A secondary outcome is to create the foundational investigation for creating a patient-reported outcomes instrument for this patient population.

## Materials and Methods

The inclusion criteria included all patients who underwent a maxillectomy surgery over 1 year prior with a surgical reconstruction or an obturator insertion for a malignant lesion between 2009 and 2020. Exclusion criteria included the lesion being non-malignant and disease recurrence within the first year. Ethics approval was granted by the Research Ethics Boards at Western University (115547). Patients reconstructed surgically were recruited from the Department of Otolaryngology—Head and Neck surgery, through the directories of the head and neck surgeons at LHSC (Dr. McNeil, Dr. Fung, Dr. Mendez, Dr. Yoo) and the private practice of Dr. Aragon for obturator patients. Participants were selected based on their access to Zoom as the study was conducted during the COVID-19 pandemic and ability to communicate their experience. Furthermore, to ensure the generalizability and reproducibility of our findings, we identified specific criteria for selecting participants based on timeline of interventions. We required that patients were included at least 1-year post-treatment, with a maximum of 10 years since their treatment. Since most functional symptoms following treatment stabilize after the 1-year period, the criteria of a 1-year minimum was established to ensure that patients would not have active treatment changes and would be on a steadier state of symptoms. Establishing a maximum time frame of 10 years was to ensure relevance to current surgical approaches and treatment protocols. To capture a representative range of treatments and functional challenges, we accounted for variations in treatment protocols that could include patients who underwent a combination of radiation, chemotherapy, and only surgery, all of which could influence functional domains.

Standards for reporting qualitative research (SRQR) reporting guidelines were followed. We analyzed 25 patients with a mean age of 68 years old (range = 56-95 years old), with most patients (14/25, 56%) being male. Most patients (15/25, 60%) were rehabilitated with an obturator, and 36% (10/25) underwent a surgical reconstruction. The most frequent tumor histology was squamous cell carcinoma (11/25, 44%) and 13/25 (52%) of patients had a stage III or IV disease. Maxillectomy defect was classified based on the Brown’s revised classification (Table 1).^8^ About 88% (22/25) of patients were included in phase I, and 20% (5/25) of patients were included in both phase I and II.

**Table 1.** Phase I and II Patient Demographics and Classification.

Patient demographics (Phase I and II)		
Number of patients, N		25
Age	Mean (range)	68 (56-95)
Sex, N (%)	Male	14 (56)
	Female	11 (44)
Tumor type, N (%) Tumor grade, N (%)	Squamous Cell Carcinoma	11 (44)
	Adenocarcinoma	2 (8)
	Adenoid Cystic Carcinoma	4 (16)
	Mucosal Melanoma	1 (4)
	Salivary Gland Carcinoma	1 (4)
	Esthesioneuroblastoma	1 (4)
	Odontogenic carcinoma	1 (4)
	Other	4 (16)
	I	3 (12)
	II	4 (16)
	III	6 (24)
	IV	12 (48)
Rehabilitation method, N (%)	Obturator Insertion Free-Flap Reconstruction Both	15 (60) 9 (36) 1 (4)
Phase II—obturator group		
Number of patients		4
Age (range)	Mean	69 (59-77)
Sex, N (%)	Male	1 (25)
	Female	3 (75)
Tumor type, N (%) Tumor grade, N (%)	Squamous Cell Carcinoma	3 (75)
	Adenocarcinoma	1 (25)
	I	2 (50)
	II	0 (0)
	III	1 (25)
	IV	1 (25)
Maxillectomy defect type^a^	Class 1	0 (0)
	Class 2a	4 (100)
	2b	0 (0)
	Class 3c	0 (0)
	Class 4	0 (0)
Phase II—Surgical Group		
Number of Patients		4
Age (range)	Mean	62 (47-75)
Sex, N (%)	Male	2 (50)
	Female	2 (50)
Tumor type, N (%) Tumor grade, N (%)	Adenoid Cystic Carcinoma	2 (50)
	Spindle cell carcinoma	1 (25)
	Other (lymphoma)	1 (25)
	I	0 (0)
	II	0 (0)
	III	3 (75)
	IV	1 (25)
Maxillectomy defect type^a^	Class 1	0 (0)
	Class 2a	2 (50)
	2b	1 (25)
	Class 3c	1 (25)
	Class 3	0 (0)

a Maxillectomy defect type was classified based on the Brown’s revised classification of maxillectomy defect.

Phase I employed open-ended questions and phase II used a modified Delphi technique. Clarifying questions were used if the patient has difficulty understanding the question (Table 2). Both phases were conducted virtually over a Zoom^®^ meeting between January 22, 2020, and June 17, 2022. Interviews were recorded and transcribed verbatim. A mixed methods qualitative approach was used.

**Table 2.** Questions Used in Phase I and II.

Phase I questions	
Question	Further clarification (if needed)
What is important in terms of your health?	What are areas of your health that make a large impact on your quality of life? Some areas that patients have previously mentioned include speech, swallowing, eating. . .etc.
How do you feel about your mouth, teeth, and throat after treatment?	How are things different from before the treatment or before your diagnosis?
Other than curing the disease, what ideally would treatment do for you?	What problem(s) is your cancer causing you that you would want relieved?
Are there any specific problems you are currently experiencing with your mouth, teeth, and throat?	
**Phase II** Questions if conversation lagged.	
**Question 1** Are there any items missing from the list that affected their functioning pre- or post-treatment	
**Question 2** Are there better ways of expressing any of the items?	
**Question 3** Would you make a different treatment choice knowing what you know now?	

### Phase I: Grounded Theory and Data Extraction

Once patients consented for the study, each patient individually underwent a 60-minute virtual interview designed to identify functional domains of interest. Patients were presented with 4 open-ended questions that were generated from a literature search on functional outcomes in head and neck cancer as well as from grounded theory methodology.^1-7,9-15^ If patients required clarification regarding the questions, a scripted clarification paragraph was read to them (Table 2).

Phase I identified the overall domains and functional outcome categories for all patients. Analysis was performed using the principles of grounded theory^13^ and NVivo software where repeating themes within patient interviews were identified using the NVivo software to categorize areas of concern for patients. As per grounded theory principles, inductive thematic analysis was used to extract small units of meaning, which were then given codes or labels. Similar codes were then grouped together to form larger, overall categories (i.e., domains). The domains were then compared to assess whether there were domains specific to patients who underwent surgical reconstruction or obturator insertion (Table 3).

**Table 3.** Functional Domains Obtained in Phase I.

Functional domains	
Obturator group Speaking difficulties Eating difficulties Dry mouth Pain Eye-related symptoms **Obturator discomfort**^a^ Social Discomfort with public eating Breathing difficulties Decreased stamina Swallowing difficulties Weight loss Concerns about public appearance Chewing difficulties Excess moisture in the mouth Feelings of numbness **Any loss of senses**^a^ Decreased appetite **Difficulty drinking** ^a^ Heart problems **Voice changes**^a^ Scratchy or sore throat **Obturator discomfort**^a^ **Nasal regurgitation**^a^ Oral hygiene	Surgical reconstruction group Speaking difficulties Eating difficulty Dry mouth Pain Teeth problems Swallowing difficulty **Trismus**^a^ Difficulty chewing Excess moisture in the mouth **Nosebleeds and nasal drip**^a^ Scratchy or sore throat **Gum-related issues**^a^ Breathing difficulty Decreased stamina Weight loss Numbness Skin changes Headaches Swelling Social discomfort with public eating Concerns about appearance Oral hygiene.

a Indicates functional domains unique to each group.

### Phase II: Focus Groups

Two focus groups were conducted based on the 2 reconstruction methods. An expert from the Centre for Education, Research, and Innovation (CERI), Western University was consulted to overview the focus group protocol. The first focus group conducted was the surgical reconstruction group and the second was the obturator group. Both groups included 4 patients each. All patients had to fall within inclusion criteria of phase I and were included based on communication skills and educational background in accordance with the Delphi technique protocol.^15^ The method of rehabilitation and ability to access technology were also considered for participant selection. Gender, cancer staging, and time since treated were factors considered to match patients as closely as possible in each group.

To refine the list of functional domains of concern from phase I into a priority list, the modified Delphi technique was implemented.^15^ Expert stakeholders, which are required by the Delphi technique, consisted of patients with maxillary cancer who underwent a maxillectomy and were reconstructed surgically or with an obturator. Being in a unique expert position relating to their experience, they were able to provide insight on the importance of the various listed functional outcomes. Once patients were identified, they were contacted by phone and consented for the study. After being consented, patients were sent the domain result list from phase I based on rehabilitation group (Table 3).

Instructions included asking the patients to review the functional domain list and numerically rank the items from most to least important in their opinion and email the ranked list back prior to the discussion. Focus group discussion for each group was 1 hour long and patients were asked to talk about their treatment experience with the goal of identifying the domains that would most significantly impact their functioning. A set of pre-approved and standardized facilitating questions were utilized by the independent facilitator if there was a lag in conversation (Table 2). To reach an agreement, patients decided to rank the domains based on how much it would affect functioning had they experienced each domain. The focus groups were concluded when the top functional domains were decided upon with over 70% agreement from the focus group participants.

### Coding Methods

Coding was done by AA as a medical student, with the expertise of AM, an otolaryngology surgeon with a background in qualitative research and coding. The coding was also reviewed by CD (DDS), to incorporate a multidisciplinary approach and help mitigate individual biases through variety of perspectives. Initially, open coding was utilized as per grounded theory methodology to establish the themes. This was followed by axial coding, where relationships between categories were established. Finally, selective coding was utilized to refine and integrate categories into these. An example of themes and categories derived from patient quotes can be seen in Appendix 1.

To ensure the comprehensiveness of the analysis, we utilized a code-recode strategy where AA revisited the data after their initial codes, with discussion with CD and AM to ensure consistency and accuracy. AA also utilized memo writing throughout the coding process, where memos were written to document emerging ideas on NVivo, to capture the evolving process of analysis. These memos were then used in peer debriefing as a bases of discussion with CD and AM. We engaged in biweekly meetings with the study group, HK, AM, AA, and CD, after the initial patient interviews where we would go over AA’s coding and ensure agreement amongst the study team.

### Note on Sample Size

In this qualitative analysis, a small sample was expected as is typical for qualitative analyses focused on exploring patient experiences. Patients were identified and contacted based on specific eligibility criteria, as previously outlined. Throughout our interviews with patients, we aimed to reach theoretical saturation point, where additional participants contribute less than 20% new information or domains. We initially reviewed data from the first 5 patients, analyzing the themes and domains that they raised. With each subsequent participant, we monitored their answers using the NVivo software for any new domains, and with each additional patient interview, there was a decrease in the number of novel insights. Saturation was reached after 12 participants, but we continued with 10 more participants in phase I to ensure comprehensive coverage of themes and variability in perspectives.

## Results

Through identifying domains in phase I, results showcased that the obturator group was uniquely affected by obturator discomfort, nasal regurgitation, loss of senses, decreased appetite, difficulty drinking, and voice changes. The surgical reconstruction group was uniquely affected by trismus, nasal bleeds or drip, and gum-related issues. Remaining domains were shared amongst the 2 groups (Table 3).

The functional domain list identified in phase I was refined in phase II. Important areas of concern were identified for each group (Table 4). For the obturator group, many of the functional domains were attributed to obturator discomfort. In the obturator group, 3 out of 4 patients underwent radiation and had significant symptoms only during treatment, such as weight loss. The top functional domains for the surgical reconstruction group were speaking difficulties, eating difficulties, dry mouth, eye-related symptoms, trismus, chewing difficulties, numbness, and social discomfort with public eating (Table 4). There were 5 functional domain items that were shared between the 2 focus groups: speaking difficulties, eating difficulties, numbness, and social discomfort with public eating (Table 5).

**Table 4.** Phase II: Top 8 Functional Domains for the Obturator and Surgical Reconstruction.

Category	Functional Domains
Shared between the surgical reconstruction and obturator group	Eating difficulties Drinking difficulties Speaking difficulties Social discomfort with public eating Numbness

**Table 5.** Functional Domains Shared Between Groups.

Surgical reconstruction group	Obturator group
Speaking difficulties	Obturator discomfort
Eating difficulties	Eating difficulties
Chewing difficulties	Drinking difficulties
Dry mouth	Speaking difficulties
Trismus	Voice changes
Numbness	Nasal regurgitation
Social discomfort with public eating	Numbness
Eye-related symptoms	Social discomfort with public eating

In the surgical reconstruction focus group, patients emphasized the importance of pre-treatment education, especially concerning the donor site effects post-resection. Additionally, several patients indicated that the most painful part of the process was the surgical recovery. Although the treatment process was difficult and challenging, patients believed that this method of rehabilitation worked the best for them. Similarly, obturator patients also unanimously agreed that they would not change their treatment course if they could. Overall, the functional domains that were ranked as the most important for both rehabilitation groups were eating, drinking, and speaking difficulties, and social discomfort with public eating. Domains unique to the obturator group were obturator discomfort, nasal regurgitation, weight loss, and voice changes. Domains unique to the surgical reconstruction group were dry mouth, trismus, chewing difficulties, and eye-related symptoms.

## Discussion

This study aimed to identify important functional areas of concern in patients undergoing maxillectomy surgery, to compare similarities and differences in these domains between patients reconstructed using surgery versus obturators, and to develop the initial phases required for the creation of a patient-reported outcomes instrument. Several factors influence the choice of reconstruction method post-maxillectomy, including the defect size, prosthesis stability, and remaining tissue positioning.^16,17^ The decision of which rehabilitation method to use is complex, and both surgical and obturator reconstructions carry their own effects on patients’ perception of importance, as it was shown by this study’s functional domains. The use of PRO’s assists in integrating the patient’s own perceptions of importance in this decision-making process.

Surgical reconstruction as a rehabilitation method post-maxillectomy has been a well-established treatment.^16-20^ Previous research demonstrated the reliability of the surgical reconstruction method,^21-23^ but when assessing whether there is a difference in overall functioning for surgical reconstruction when compared to obturator insertion, there are limited studies in the literature that were able to demonstrate a significant difference that favors either rehabilitation method over the other.^16,17,19,20,23^ One such study, a meta-analysis performed by Cao et al,^24^ demonstrated that patients who underwent surgical reconstruction showed better word intelligibility (*P* = .004) and mastication (*P* = .002) compared to those who had obturator prosthesis. However, no difference was shown for speech intelligibility or nasalance in the systematic review, and in a best evidence synthesis, no difference was found for speech, pain intensity, saliva production, maximum mouth opening, or depression.^24^ Previous studies have been unclear regarding what domains affect the functioning of these patients the most after they undergo their rehabilitation.^16^ The importance of patient education prior to surgery in association with engagement in recovery has also been previously emphasized.^25^ Educating patients regarding their treatment course includes understanding what affects patient’s functioning with each rehabilitation choice. There are limited studies that assess the longitudinal impacts of rehabilitation choices on overall functioning. Since both rehabilitation methods are commonly used and result in reliable treatment outcomes, knowing the similarities and differences creates a way for patients and healthcare professionals to assess which option would limit each individual patient’s function the least and offer further autonomy to patients.

This study showcased that although there are several similarities between the obturator and surgical reconstruction groups, there are unique domains relevant to each group that affects functioning in unique ways (Table 4). Patients had shared difficulty that includes speaking and eating, which are essential skills that are used for daily functioning. When looking at unique functional domains, the obturator focus group had difficulties largely related to the obturator placement itself. In phase II, the obturator focus group discussion showcased the importance of patient education as it was valued by the patients and significantly assisted them in evaluating their options prior to treatment and in their recovery. The patients in this study had access to care from a prosthodontist in private practice and many reported satisfaction with the dental care they received, which contributed to their ability to adapt to wearing an obturator. For some patients, preoperative education would have included knowing that their obturator may need a series of adjustments by a prosthodontist to achieve comfort, function, and esthetics. The obturator discomfort resulted in eating, drinking, and speaking difficulties, as well as voice changes.

In comparison, preoperative education for the surgical reconstruction group would have facilitated discussion regarding the potential of decreased functioning related to the site of surgical reconstruction. The surgical reconstruction group had difficulties related to the functional use of sites affected by the reconstruction, such as trismus and nasal bleeds/drips. The adjuvant use of radiation for some patients may have resulted in worsening functional domains, such as in the case of trismus following radiation. Patients’ ability to adapt to functioning, whether it is with an obturator or with surgical reconstruction, is facilitated when they are educated in advance on what to expect. This study established a baseline from which specific functional domains related to rehabilitation groups can be compared. This information enables patients to make an informed decision and play a role in their treatment process.

Limitations of the study include difficulty in patient recruitment due to the small sample size of patients who underwent a maxillectomy. The limited patient population size resulted in a decreased ability to match patients exactly based on age and time since treatment. This also results in the need to rely on patients that are years post treatment, sometimes dating back to 2009. This selection bias may have affected the results as patients who had more aggressive disease may have passed away, thus potentially resulting in increased participation of patients with less severe disease. Most patients generally adapt and can return to their original or near original functioning 12 months post-surgery.^26^ As such, patient-reported outcomes may have differed based on time since treatment. Additionally, several patients were unable to participate in virtual interviewing. Due to COVID-19 restrictions and given the older age of the patient population, limited access to technology affected the ability to participate. Grounded theory also includes researcher bias. Coding decisions and theme interpretation can be influenced by the researcher themselves who is doing the coding, as well as preconceptions that the research team may have. To mitigate this potential bias in this study, the study team had frequent meetings about the coding progress, with transparency regarding the themes as well as utilizing open and axial coding. Code-recode approach, peer debriefing, and memo tracking were also utilized to strengthen the reliability of our results. Lastly, it should be noted that quality of maxillectomy reconstruction, either with a flap or obturator can be variable depending on the circumstance. In this study, the final quality of the reconstruction was not considered during patient recruitment or the coding process and thus this study did not capture how this may impact functional domains in either group.

## Conclusion

The functional domains that were identified in our study will allow patients to receive standardized information to assist in making an informed decision during their maxillectomy preoperative treatment planning. Other factors identified, such as the ongoing need for prosthesis adjustments, the potential increased cost that may be required to address ongoing oral health needs, previous experience wearing a prosthesis, recovery time, decreased function of surgically reconstructed areas, and treatment time may also influence a patient’s treatment decision.

This study was able to identify unique domains that impact maxillectomy patients based on the rehabilitation method of a surgical reconstruction or an obturator insertion. More similarities than differences exist between the 2 groups, but it is the differences which may be the most useful in patients’ decision-making process. There is significant value in understanding which domains impact a patient’s functioning, especially when the results could impact choice of rehabilitation. The findings from this study can assist healthcare practitioners in continuing to view patients holistically and guiding patients regarding their treatment course. An awareness of patients’ functional domains of importance will allow provision of support within multidisciplinary teams that minimize the impact of treatment and improve patient survivorship. Patient education also allows for further autonomy and active participation of patients in their treatment.

## Appendix 1

**Table A1.** Example of Themes and Categories Derived From Patient Quotes.

Patient	Quote	Theme	Category
A	“There are things I can’t eat. For example, I can’t eat nuts anymore. I have sometimes trouble chewing meat a bit”	Inability to eat certain foods	Eating difficulty
B	“. . .so when I’m eating, I have to be very careful or I’ll bite on my lip, which is kind of in the way of my teeth sometimes”	Physical constraint of eating	Eating difficulty
C	“Eating, eating is difficult. . .” “I just finished a sandwich; I couldn’t bite into a soft bread sandwich. I had tomato and sweet onion on it, miracle whip. I took a bite and my obturator flipped right out. If I try to bite on something, unless I bite right on the side where my natural teeth remain on the very side of my mouth, I can’t bite into anything”	Loss of control during eating	Eating difficulty
